# Safety and Tolerability of the Acute Ketamine Treatment in Treatment-Resistant Depression: Focus on Comorbidities Interplay with Dissociation and Psychomimetic Symptoms

**DOI:** 10.3390/ph16020173

**Published:** 2023-01-24

**Authors:** Adam Włodarczyk, Alicja Dywel, Wiesław Jerzy Cubała

**Affiliations:** Department of Psychiatry, Faculty of Medicine, Medical University of Gdansk, 80-214 Gdansk, Poland

**Keywords:** ketamine, treatment-resistant depression, major depression, antidepressants, dissociation, psychosis, comorbidity, safety

## Abstract

There is evidence for ketamine use in treatment-resistant depression (TRD). Several safety concerns arise regarding adverse drug reactions in specific subpopulations. The aim of this study was to investigate the safety of intravenous ketamine treatment in relation to dissociative and psychotic measures in TRD inpatients with Major Depressive Disorder (MDD) and Bipolar depression (BP) with comorbidities. In total, 49 inpatients with MDD or BP were treated with ketamine following the registered naturalistic observational protocol in a tertiary reference unit for mood disorders (NCT04226963). This dataset represents an intermittent analysis of an observational study performed for interim modeling of observational learning. The observations were applied to the inhomogeneous TRD population in a single site with no blinding and were limited to acute administration. The presence of epilepsy was significantly associated with an elevation in the BPRS over time (*p* = 0.008). Psychotic symptomatology with BPRS scores for comorbid conditions excluding epilepsy turned out to be insignificant (*p* = 0.198) regardless of the diagnosis. However, for a subgroup of patients with epilepsy (*n* = 6), a substantial fluctuation was seen across all administrations in the time course of the study. The study results contribute to the literature on the safety and tolerability profile of CNS adverse drug reactions in short-term treatment with intravenous ketamine as an add-on intervention to current standard-of-care psychotropic medication in TRD-MDD and TRD-BP inpatients with comorbidities. The careful consideration of comorbidities and concomitant medication is needed with ketamine administration along with close-clinical supervision at every visit.

## 1. Introduction

There have been recent developments in rapid-acting antidepressant medication use in treatment-resistant depression (TRD) with esketamine nasal spray as an add-on to antidepressants approved for treatment-resistant depression provide evidence for ketamine use in Major Depressive Disorder (MDD) and Bipolar Disorder type I (BP) for rapid remission of depressive symptoms, but with concerns about its safety and tolerability [1].

One of the major issues is the risk of adverse events associated with dissociative symptomatology [2]. There is some evidence for dissociative symptoms as the predictor of response in TRD both TRD-MDD and TRD-BP; however, it is limited to very few studies [3]. However, even more studies, including our own, show that there is no relationship between dissociative symptomatology and depression outcome. Overall, little is known about the course of dissociative symptomatology regarding ketamine use in affective disorders [4]. Dissociative states involve symptoms of gaps in memory, out-of-body experiences, and depersonalization, derealization, and identity disturbance [5]. This phenomenon is associated with ketamine administration [6]. Dissociative symptoms cause a wide spectrum of phenomena; however, per the methodological guidance, Clinician-Administered Dissociative States Scale (CADSS) and Brief Psychiatric Rating Scale (BPRS) with positive symptoms subscale (BPRS+) are used to represent the overall intensity of the dissociative and potential treatment-emergent psychotic symptomatology [2,3,4,7,8,9,10,11]. 

Scarce data are available on ketamine use in TRD patients with comorbidities [12]. In line with the measures of depression symptomatology (MADRS), safety measures (CADSS, BPRS) are used to assess dissociation and psychotic phenomena.

This study aimed to assess the safety and tolerability profile of intravenous ketamine in the course of eight administrations in inpatients with TRD as related to the clinical characteristics.

## 2. Results

Sociodemographic characteristics are given in Table 1. Out of 49 patients included in our study, 21 of them had comorbidities. All the patients were medically stable, including those significantly affected by illness continued current medication during ketamine treatment. A given type of disease was set for the analyses performed as an inter-object factor. The detailed results of the analyses are presented in Table 2 and Table 3.

The post-dose maximum levels of both the CADSS and the BPRS scores declined with subsequent ketamine infusions. In every infusion after rising up to 30 min post ketamine intake, declining to ‘absent’ in maximum 60 min post-infusion, except the epilepsy subgroup. During the follow-up visit (one week after last ketamine administration) neither CADSS, nor BPRS scores were identified as present.

The CADSS scores (Table 2), and the BPRS scores (Table 3) were analyzed for simple effects with Bonferroni correction.

The interaction effect is demonstrated collectively (Table 2 and Table 3) presenting main effect and simple effect for interaction.

The main effect for CADSS over time turned out to be significant (CADSS scores increased) only for the diagnosis of hyperlipidemia, F (4.58) = 5.04; *p* < 0.001; η2*p* = 0.10, similar to interaction (referring to the results from Table 2 where the data from interaction between hyperlipidemia and CADSS are presented). After the Bonferroni correction, simple effects for the CADSS for people without hyperlipidemia (*n* = 40) turned out to be insignificant, F (7.40) = 1.89; *p* = 0.09; η2*p* = 0.25 as in the case of hyperlipidemia diagnosis (*n* = 9), F (7.40) = 1.92; *p* = 0.09; η2*p* = 0.25, also appeared insignificant.

Epilepsy was the only diagnosis significantly associated with changes in the BPRS over time. The main effect for BPRS turned out to be significant, F (3.96) = 8.53; *p* < 0.001; η2*p* = 0.20, similar to the interaction (referring to the results from Table 3 where the data from interaction between epilepsy and BPRS are presented). After considering the Bonferroni correction, simple effects for the BPRS for people without epilepsy (*n* = 43) turned out to be insignificant, F (7.28) = 1.53; *p* = 0.198; η2*p* = 0.28, while significant for people with epilepsy (*n* = 6), F (7.28) = 3.54; *p* = 0.008; η2*p* = 0.47. In patients with epilepsy, significant effects occurred for measurements after the infusion of 1st F (1.34) = 10.41; *p* = 0.003; η2*p* = 0.23, 6th F (1.34) = 12.35; *p* = 0.001; η2*p* = 0.27, and 8th F (1.34) = 18.05; *p* < 0.001; η2*p* = 0.35, with higher results (BPRS scores increased) obtained for patients with epilepsy.

This study demonstrates favorable safety and efficacy profile for treatment with intravenous ketamine for TRD with comorbidities. Variations in CADSS and BPRS values across IV ketamine treatment in TRD are seen at treatment administration with no sequelae exceeding 30 min post-infusion. Exacerbation was observed after the infusion of 1st, 6th, and 8th, only for patients with epilepsy subgroup (*n* = 6).

## 3. Discussion

This post hoc analysis points out the favorable safety and tolerability profile of short-term ketamine use in TRD subjects with both MDD and BP with concomitant psychotropic medication and comorbidities as the potentially confounding factor that may be associated with the manifested safety and tolerability profile. The pronounces effect presented with patients with concomitant epilepsy.

There is a need for novel approaches for both TRD-MDD and TRD-BP in people with somatic comorbidities, as they may present distinct efficacy/safety/tolerability profiles than otherwise healthy participants represented in the majority of studies [13,14], i.e., depression is the most frequent comorbid psychiatric disorder in epilepsy [15]. Ketamine is known for its psychomimetic-adverse event potentials [2,16]. The symptoms captured by the CADSS and BPRS questionnaires could be reflecting neuroplasticity stemming from the engagement of primary and secondary visual areas as a consequence of the dissociative and psychomimetic phenomena of ketamine [15]. However, there is a safety concern regarding treatment with ketamine with comorbidities, while also little is known about N-methyl-D-aspartate receptor (NMDA) antagonists for refractory seizures, outcomes were poorly documented in the majority of the studies [16]. Overall, this study is in line with esketamine trials [6,13,14,17], as it shows to produce no harm with esketamine treatment, and all of the patients experienced any persistent dissociative or psychotic symptoms during the follow-up visit. Although it must be noted that real-world pharmacovigilance analysis of the FDA Adverse Event Reporting System database detected new, unexpected signals, with serious adverse events, clearly indicating that further studies on ketamine/esketamine are needed [18,19]. We have found in the literature that irreversible changes reported in the rat brain, called ‘Olney’s lesions’, developed after ketamine infusion [20]. However, the human brain metabolism is different from rat brain metabolism, therefore such changes may not appear in human brain tissue [20]. There is also evidence that short-term exposure of gamma-aminobutyric acid neurons to high doses of ketamine led to a significant loss of differentiated cells in one study, and non-cell-death-inducing concentrations of ketamine (10 μg/mL) may still establish long-term transformation of dendritic arbor in differentiated neurons [21]. The study by Vutskits et al. [22] also demonstrated persistent (>24 h) administration of ketamine at concentrations as low as 0.01 μg/mL can interfere with the maintenance of dendritic arbor architecture. These results raise the possibility that persistent exposure to subanesthetic doses of ketamine, could still damage neuronal maintenance and development, without affecting cell survival. Further studies are needed to explore that matter, not only due to psychomimetic symptomatology present more frequently in people with epilepsy, but also for the possible long-range side-effect of possible ketamine treatment. 

Although classical antidepressants are thought to be safe to use in epilepsy, there is very limited evidence demonstrating a significant effect of antidepressants on depressive symptoms in epilepsy [23,24], and no data about TRD-MDD treatment in this population. Ketamine has both pro and anti-convulsive properties; however, apart from some sparse data published [25] not long after the FDA registration of Ketalar, there are no controlled human studies of the effect of ketamine in epilepsy in anesthetic or subanesthetic doses [25]. Moreover, recently, ketamine has been successfully used in the treatment of status epilepticus, and it has been suggested item has some neuroprotective properties [26]. The occurrence of various psychiatric disorders in people with epilepsy is high, with psychoses affecting 2–9% of patients [27]. In another cross-sectional study by Klaudee et al. (2019) [28] on the Thai population, from a total of 170 patients with epilepsy 43 (25.3%) fulfilled diagnostic criteria for one or more psychiatric disorders where psychotic disorders were 8.2%. Another study found that apart from comorbid mood and anxiety disorders, patients with comorbid epilepsy with an interictal dysphoric disorder were also more likely to suffer from psychotic disorder [29]. The symptomatology of mood disorders in epilepsy is often atypical, pleomorphic, and fails to fulfill DSM diagnostic criteria, thus the treatment of mood disorders in epilepsy often requires a non-standard, individual approach [30].

There are several limitations to influence the findings of our study. First, the number of participating subjects was relatively few, and the findings may be implicated due to the fact that the study is likely to be underpowered. Second, the research was performed as a single-site study, there was no treatment blinding, during this observational protocol study. The observations applied to treatment-resistant patients and included both patients with unipolar and bipolar depression. Finally, The CADSS has limitations as a tool to measure the acute effects of ketamine infusions [30]. Additionally, no CADSS assessment was obtained only once post-dose (30 min post-dose) without a few measurement time points, so we could not establish the precise time course of either the peak dissociative symptoms or their resolution. The primary strength of this study is the report on a specific population of interest that answers the question of safety profile in subjects presenting comorbidities and generates a hypothesis for further research. Another limitation is whether it was antiepileptic medication rather than the diagnosis of epilepsy which led to the increases in BPRS in these participants—which stands in line with our previous findings [31,32]. Post hoc analysis from the observational registry cannot be generalized nor produce causative observations. Thus, results require cautious interpretation per biases specific for decisions biased studies.

The presented study provides information that in ketamine use, careful consideration of comorbidities and concomitant medication is needed, while ketamine administration close-clinical supervision is necessary at every visit. Somatic comorbidity may impact dissociative symptomatology, and psychotic symptoms must be taken into consideration in planning treatment with TRD patients with epilepsy.

## 4. Materials and Methods

### 4.1. Participants

The sample selection for this study has been described in detail elsewhere [31,32,33,34]. Briefly, the study population comprises subjects enrolled in a naturalistic safety and tolerability registry protocol for ketamine infusions in TRD. This dataset represents the intermittent analysis of an observational study performed for the interim modeling of observational learning. The analyses are set per predefined and equally spaced annual increments for a study recruitment period of 24 months with a population of 49 TRD subjects estimated for inclusion. All subjects participated in the active ketamine administration followed by one week follow-up period, in a study recruiting subjects at the site for 24 months. Inpatients diagnosed with a depressive episode in the course of major depression, recurrent depression, or bipolar affective disorder were involved. Patients were interviewed by a clinician psychiatrist to establish the diagnosis according to the Diagnostic and Statistical Manual of Mental Disorders (DSM-5) criteria determined using the Mini International Neuropsychiatric Interview (MINI) [35]. The MINI interview is a short structured clinical interview that enables researchers to make diagnoses of psychiatric disorders according to DSM-5. 

All participants met the criteria for TRD, defined as an inadequate response to 2 or more antidepressants of different categories (assessed by the Massachusetts General Hospital Antidepressant Treatment Response Questionnaire—ATRQ) [36] in the course of treatment of that particular episode. ATRQ is a clinician-assisted questionnaire that examines a patient’s antidepressant treatment history using specific anchor points to define the adequacy of both the dose and duration of each antidepressant course, as well as the degree of symptomatic improvement obtained with each course. Bipolar TRD was defined as a clinically unsatisfactory response following at least two trials of dissimilar medicinal treatments in adequate doses and durations, within a specific phase of bipolar illness [37]. The study followed the rule single-patient and single-rater (the same patient was examined by the same clinician in all of the scales). 

Only medically stable, able to communicate and provide consent, adult inpatients aged 18–90 were enrolled to study. Some patients were significantly affected by illness; however, all of the patients continued current medication during ketamine treatment without any changes to the substance and/or dosage. The detailed description of antiepileptic medication in patients with epilepsy is presented in Table S1. The exclusion criteria included a history of uncontrolled medical conditions, a previous adverse reaction to ketamine, active substance use, pregnancy, or breastfeeding. Comorbidities were defined by the patient’s self-reported medical history with corroborated full-passed medical records. Comorbidities relevant to this study group population were arterial hypertension, diabetes mellitus, hyperlipidemia, post-stroke, and epilepsy (Table 1).

### 4.2. Study Design

All patients continued baseline antidepressants, as well as treatment of chronic diseases during ketamine administrations. This study used an observational design with the administration of eight ketamine intravenous infusions over 4 weeks. Ketamine was dosed at 0.5 mg/kg based on the patient’s actual body weight and infused intravenously over 40 min. Any significant adverse effect was also monitored, either by safety measurements including vital signs or per safety observation by clinical investigators. The electrocardiogram (ECG) was carried out before every second infusion and one week after the last ketamine infusion. One week after the last infusion laboratory tests, ECG, all mentioned scales were performed.

Safety monitoring was performed every 15 min before, during, and after the infusion until an hour and a half after the infusion. This monitoring included the assessment of vital signs (i.e., heart rate, body temperature, respiratory rate, blood pressure, oxygen saturation) as well as the administration of mental status examinations, including assessments by the BPRS and the CADSS, to determine the presence of psychotic and/or dissociative symptoms. Any other significant adverse effects were also monitored and recorded. Psychometric assessments by the MADRS and the YMRS were administered before the 1st, 3rd, 5th, and 7th infusions as well as one week after the last infusion. An ECG was carried out before every second infusion and one week after the last ketamine infusion.

### 4.3. Safety and Tolerability Measures

During the screening patients were rated by the clinician using Montgomery–Åsberg Depression Rating Scale (MADRS) [38], Young Mania Rating Scale (YMRS) [39], Columbia–Suicide Severity Rating Scale (C-SSRS) [40], The Clinician-Administered Dissociative States Scale (CADSS) [5], Brief Psychiatric Rating Scale (BPRS) [41] scales. The MADRS is a clinician-assessed measure of depression severity in clinical trials and was developed to provide a measure of depression severity for use in antidepressant response studies. For this purpose, 10 items were selected based on their ability to detect depression change. The YMRS assesses hypomanic/manic symptom severity. It is an 11-item clinician-administered scale, with a total score range of 0 to 60 (≤12 indicates remission, 13–19—minimal symptoms, 20–25—mildly manic, 26–37—moderately manic, and 38–60—severely manic), while the C-SSRS is partially structured clinical history assessing the severity of suicidal thoughts, their intensity, and suicidal behavior. The CADSS was chosen for analysis as it is the most widely used instrument employed in previous mood disorder studies to assess the acute psychoactive effects of ketamine [7]. The CADSS includes a 19-item scale used to evaluate the patient’s answers (subjective items) and an 8-item scale used by a trained physician to assess the patient’s responses during ketamine intake (objective items). The subjective items include three components: depersonalization, derealization, and amnesia. The BPRS is an 18-item rating scale used to assess a range of psychotic and affective symptoms based on both observation of the subject and the subject’s own self-report. A variant of the BPRS is the four-item BPRS+, which considers the positive symptoms of suspiciousness, hallucinations, unusual thought content, and conceptual disorganization. The BPRS and the BPRS+ are used to assess treatment-emergent psychotic symptoms. In both tests, each symptom is rated on a scale from 0 to 6, where 1 is “not present” and 6 is “extremely severe” (the score of 0 represents a not assessed item). To demonstrate the CADSS and BPRS fluctuations across treatments, CADSS and BPRS scores taken 30 min post drug administration were analyzed.

A subject was defined as a responder at a given time point if the percent improvement from the baseline total MADRS score was at least 50% and the subject did not remit. The patient was defined as a remitter at a given time point if the total MADRS score was ≤10 points [42]. The final three groups (responders, remitters, and nonremitters) were determined by MADRS score at the follow-up visit, one week after the last ketamine infusion. The study outcome measure is defined per a MADRS score.

### 4.4. Statistical Analysis

The analyses were conducted using statistical software the IBM SPSS Statistics 25.0. To determine the differences between responders, remitters, and non-responders for sociodemographic variables and the occurrence of diseases and treatment, frequency analyzes were carried out with Fisher’s exact test. To determine the differences between measurements, mixed model ANOVA was used where the within-subject effect was the repeated measurements in CADSS or BPRS scores and the between-subject effect was the difference between different comorbidity groups. The interactions between two factors (within-subject and between-subject) were calculated. Analysis of quantitative variables was carried out by the Kruskal-Wallis test, employed to verify statistically significant differences between the groups and the Cramer’s V as a measure of association between two nominal variables. 

The medium-term rate of change of the analyzed variables was calculated using chain indexes—the harmonic average of all chain indexes was calculated. In the chain-linking method, an index of a period is calculated referred to the period immediately before that period (known as the “link index”), and the indices of two consecutive periods are multiplied in series to find the index (known as the “chain index”) In the chain-linking method, the chain index is calculated by multiplying the link indices of two consecutive periods in series, and there are two types of periods to link (linking points) [43,44]. The chain index approach identifies cases where the rate of change differs across comorbidities, and that the linear mixed model can be used to facilitate interpretation of chain index results. Based on the medium-term rate of change, the rate of change was calculated for a given variable and the relationships between the dynamics of change between variables were determined. α = 0.05 was adopted as the level of significance for this analysis. Due to the small study sample and many hypothesis tested, the Bonferroni correction was introduced to avoid making Type I error. The Bonferroni correction was used for tests since multiple comparisons were tested in such a way that the alpha value (*p*-value) was adjusted by the number of comparisons being performed. *p*-values presented in the current report reflect those obtained from a post hoc Bonferroni analysis.

The study was carried out in accordance with the latest version of the Declaration of Helsinki. For each participant, written consent was obtained after the procedures had been fully explained. The study recruitment procedures were approved by the Ethics Research Committee of the Institution. The study population comprises MDD and BP subjects treated with ketamine registered in the naturalistic observational protocol of the tertiary reference unit for mood disorders (NCT04226963).

## 5. Conclusions

The study results contribute to the literature on the safety and tolerability profile of CNS adverse drug reactions in short-term treatment with intravenous ketamine as an add-on intervention to current standard-of-care psychotropic medication in TRD-MDD and TRD-BP inpatients with somatic comorbidities. Showing contribution for practitioners for treating inpatients representing real-life population per somatic comorbidities. Moreover, chronic, disabling TRD may be confounded with somatic drug adverse effects. Careful consideration of comorbidities and concomitant medication is needed with ketamine administration along with close-clinical supervision at every visit. Larger long-term studies are needed to replicate the safety observation for no long-term psychomimetic side effects and to facilitate causative effect.

## Figures and Tables

**Table 1:** Demographic and clinical variables.

			*N*	Responder	Remitter	Non Responder	*p-Value*	V
Male sex	(%)		21 (42.9)	6 (66.7)	2 (25.0)	13 (40.6)	0.229	0.26
Female sex	(%)		28 (57.1)	3 (33.3)	6 (75.0)	19 (59.4)		
Mean age, in years			50.02	53.11	42.88	50.94	0.336	0.00
Ketamine treatment for	MDD		35 (71.4)	8 (88.9)	5 (62.5)	22 (68.8)	0.475	0.19
	BP		14 (28.6)	2 (11.1)	5 (37.5)	7 (31.2)	0.485	0.18
Comorbidity							0.104	0.31
	(no. of comorbidities)	1	21 (42.9)	6 (66.7)	2 (25.0)	13 (40.6)		
		2	10 (20.4)	2 (22.2)	1 (12.5)	7 (21.9)		
		3	4 (8.2)	1 (11.1)	2 (25.0)	1 (3.1)		
	Arterial hypertension		16 (32.7)	6 (66.7)	3 (37.5)	7 (21.9)	0.037	0.37
		BP	4 (8.2)	1 (11.1)	2 (25.0)	1 (3.1)	0.052	0.66
		MDD	12 (24.5)	5 (55.6)	1 (12.5)	6 (18.8)	0.177	0.33
	Diabetes mellitus		3 (6.1)	1 (11.1)	2 (25.0)	0 (0)	0.021	0.39
	Hyperlipidemia		9 (18.4)	3 (33.3)	1 (12.5)	5 (15.6)	0.545	0.19
	Post-stroke		3 (6.1)	1 (11.1)	0 (0)	2 (6.3)	0.731	0.14
	Epilepsy		6 (12.2)	0 (0)	3 (37.5)	3 (9.4)	0.060	0.36
	Other		16 (32.7)	2 (22.2)	1 (12.5)	13 (40.6)	0.330	0.24
Coexisting treatment	TCA		8 (16.3)	1 (11.1)	1 (13.5)	6 (18.8)	1.000	0.09
	SSRIs		23 (46.9)	5 (55.6)	2 (25.0)	16 (50.0)	0.413	0.20
	SNRIs		11 (22.4)	2 (22.2)	2 (25.0)	7 (21.9)	1.000	0.03
	Other ADTs:						0.749	0.14
	(no. of other ADTs taken)	1	15 (30.6)	4 (44.4)	2 (25.0)	9 (28.1)		
		2	3 (6.1)	0 (0)	1 (12.5)	2 (6.3)		
	Antipsychotics						0.806	0.15
	(no. of antipsychotics taken)	1	12 (24.5)	2 (22.2)	1 (12.5)	9 (28.1)		
		2	5 (10.2)	0 (0)	1 (12.5)	4 (12.5)		
	Mood stabilizers						0.348	0.29
	(no. of mood stabilizers taken)	1	15 (30.6)	2 (22.2)	4 (50.0)	9 (28.1)		
		2	6 (12.2)	1 (11.1)	0 (0)	5 (15.6)		
		3	1 (2.0)	0 (0)	1 (12.5)	0 (0)		

Kruskal-Wallis *H* test, *p* ≤ 0.05; remitter group did not include the responders group; Cramer’s V are significant at the *p* = 0.05 level. *N*, sample size; *p*, probability value; V, Cramer’s V; MDD, major depressive disorder; BP, bipolar disorder; TCA, other tricyclic antidepressant; SSRIs, selective serotonin reuptake inhibitors; SNRIs, selective serotonin-noradrenaline reuptake inhibitors; ADTs, antidepressants.

**Table 2:** The interaction effect of comorbidity on CADSS scores.

Comorbidities	*F*	*df*	*p*
comorbidity	1.09	13.55	0.36
arterial hypertension	0.73	4.40	0.58
diabetes	0.64	4.43	0.65
hyperlipidemia	2.35	4.58	0.04
post-stroke	0.90	4.36	0.47
epilepsy	1.66	4.42	0.15
other	0.60	4.51	0.68

**Table 3:** The interaction effect of comorbidity on BPRS scores.

Comorbidities	*F*	*df*	*p*
comorbidity	0.99	9.94	0.46
arterial hypertension	0.83	3.44	0.49
diabetes	1.27	3.43	0.29
hyperlipidemia	0.33	3.46	0.83
post-stroke	0.87	3.39	0.47
epilepsy	7.37	3.96	<0.001
other	0.61	3.44	0.63

## Data Availability

The datasets used and/or analyzed during the current study are available from the corresponding author on reasonable request.

## Supplementary Materials

The following supporting information can be downloaded at: https://www.mdpi.com/article/10.3390/ph16020173/s1, Table S1: Antiepileptic treatment in patients with epilepsy.
