# Supplementary material for: Safety and Tolerability of the Acute Ketamine Treatment in Treatment-Resistant Depression: Focus on Comorbidities Interplay with Dissociation and Psychomimetic Symptoms
Source: Pharmaceuticals (Basel). 2023 Jan 24;16(2):173. doi: 10.3390/ph16020173 (PMC9966368; doi:10.3390/ph16020173)
Supplement: Supplementary file 1 [file pharmaceuticals-16-00173-s001.zip › pharmaceuticals-2176220-supplementary.pdf]

Supplementary File

Table S1. Antiepileptic treatment in patients with epilepsy.

**Patient no.**	**Sex**	**Age**	**Age of epilepsy onset**	**Medication taken**	**Dose (milligrams daily)**
1	M	53	47	levetiracetam	750
2	F	38	3	ethosuximide	1000
3	F	48	42	levetiracetam	750
4	M	25	6	pregabaline	450
5	F	40	37	levetiracetam	500
6	M	41	29	pregabaline	300
